# The effect of tenofovir on renal function in HIV-positive pregnant women

**DOI:** 10.7448/IAS.17.4.19694

**Published:** 2014-11-02

**Authors:** Stuart Flanagan, Lynne Barnes, Jane Anderson, Tristan Barber

**Affiliations:** 1 Department of Sexual Health and HIV medicine, Homerton University Hospital, London, UK; 2 Department of Obstetrics and Gynaecology, Homerton University Hospital, London, UK

## Abstract

**Introduction:**

Tenofovir is a commonly used component of antiretroviral therapy (ART) to reduce vertical transmission of HIV. Although systematic review of tenofovir use in pregnancy concluded it to be low risk for foetal abnormalities [1], data is limited on its impact on renal function in pregnant women. A recent South African study [2] concluded that renal dysfunction in HIV-infected pregnant women is significantly less common than in other HIV-infected adults, however there is currently no UK data. We aimed to investigate the effect of tenofovir on renal function in HIV-1 positive pregnant women in a UK clinic.

**Methods:**

We retrospectively analyzed data on renal function in pregnancy from a cohort of women attending a busy inner city London antenatal clinic. All women were screened for renal function throughout pregnancy via serum creatinine and estimated glomerular filtration rate (eGFR) calculated using modification of diet in renal disease (MDRD) and corrected for ethnicity.

**Results:**

Ninety-seven HIV-1 positive women were registered at Homerton Hospital antenatal service of a total of 105 pregnancies between January 2010 and September 2013. Tenofovir was prescribed in 71/105 pregnancies (67.6%). Of the 71 pregnancies, 41 were prescribed tenofovir pre-conception (57.7%). Of the pregnant women who started tenofovir in pregnancy, 21/31 (67.7%) were initiated before week 24 of pregnancy, in line with British HIV association (BHIVA) guidelines [3]. There was no deterioration in median serum creatinine or decline in eGFR in women prescribed tenofovir during pregnancy. At six weeks after delivery, in the 42 women who continued tenofovir therapy and had eGFR measured, one woman had eGFR=60, all others eGFR >90 (Table 1).

**Conclusions:**

Consistent with current guidelines and experience, this study shows tenofovir did not cause decline in renal function in pregnancy in our cohort of HIV-1 positive women, whether started during pre-conception or during pregnancy. More evidence should be prospectively collected looking at effects of tenofovir on other measures of tubular renal function in pregnancy such as proteinuria and protein-creatinine ratio.

**Table 1:** Renal function during pregnancy

Gestation (weeks)	No. (% of those prescribed tenofovir)	Median serum creatinine (Range)	eGFR>90 (%)	eGFR<90
12	45 (100%)	55 (46–64)	100	0
20	44 (73.3%)	53 (44–62)	100	0
24	47 (75.8%)	53 (44–65)	100	0
28	54 (79.4%)	54 (43–70)	100	0
32	54 (76.0%)	55 (41–69)	100	0
36	58 (81.6%)	56 (46–82)	99.98%	0.017%
6 weeks Postnatal	42 (63.6%)	60.5 (45–81)	99.97%	0.024%

## References

[1] Wang L, Kourtis AP, Ellington S, Legardy-Williams J, Bulterys M (2013). Safety of tenofovir during pregnancy for the mother and fetus: a systematic review. Clinical Infectious Diseases Dec.

[2] Myer L, Kamkuemah M, Kaplan R, Bekker LG (2013). Low prevalence of renal dysfunction in HIV-infected pregnant women: implications for guidelines for the prevention of mother-to-child transmission of HIV. Tropical Medicine & International Health Nov.

[3] Management of HIV infection in pregnancy women BHIVA Guidelines 2012.

